# Non-Coding Variants in Cancer: Mechanistic Insights and Clinical Potential for Personalized Medicine

**DOI:** 10.3390/ncrna7030047

**Published:** 2021-08-02

**Authors:** Marios Lange, Rodiola Begolli, Antonis Giakountis

**Affiliations:** 1 Department of Biochemistry and Biotechnology, University of Thessaly, Biopolis, 41500 Larissa, Greece; mlangke@uth.gr (M.L.); rbegkolli@uth.gr (R.B.); 2 Institute for Fundamental Biomedical Research, B.S.R.C “Alexander Fleming”, 34 Fleming Str., 16672 Vari, Greece

**Keywords:** cancer, non-coding variability, SNPs, CNVs, lncRNAs, miRNAs

## Abstract

The cancer genome is characterized by extensive variability, in the form of Single Nucleotide Polymorphisms (SNPs) or structural variations such as Copy Number Alterations (CNAs) across wider genomic areas. At the molecular level, most SNPs and/or CNAs reside in non-coding sequences, ultimately affecting the regulation of oncogenes and/or tumor-suppressors in a cancer-specific manner. Notably, inherited non-coding variants can predispose for cancer decades prior to disease onset. Furthermore, accumulation of additional non-coding driver mutations during progression of the disease, gives rise to genomic instability, acting as the driving force of neoplastic development and malignant evolution. Therefore, detection and characterization of such mutations can improve risk assessment for healthy carriers and expand the diagnostic and therapeutic toolbox for the patient. This review focuses on functional variants that reside in transcribed or not transcribed non-coding regions of the cancer genome and presents a collection of appropriate state-of-the-art methodologies to study them.

## 1. Introduction

Cancer specific regulation of transcription is manifested through ectopic activity of proximal and/or distal Regulatory Elements (REs) [1,2,3,4,5]. REs are divided into proximal or *cis*-acting regulatory elements (CREs), such as promoters, and distal or *trans*-acting regulatory elements (TREs) comprising of enhancers that establish physical contact with the former via long range 3D chromatin loops [6,7]. Given their proximity to transcriptional start sites, promoters predominantly function in a directional manner with regards to transcript orientation [8]. In contrast, enhancers can be located upstream or downstream of the target gene as well as within intronic regions and they can operate from a distance and in a bidirectional fashion [9]. Promoter–enhancer communication is mainly established in the form of intrachromosomal chromatin loops, while in some rare occasions enhancers may establish interchromosomal interactions with promoters [10,11]. Distinct epigenetic marks for each regulatory element facilitate dynamic chromatin accessibility and nucleosomal repositioning, which in turn dictate transcriptional status of the target locus.

More specifically, nucleosomes in enhancer and promoter elements are decorated with histone acetylation H_3_K_27_Ac, which generally marks open chromatin, while histone methylation such as H_3_K_4_Me_3_ is indicative of active promoters [8]. Epigenetic modifications of enhancer loci can be subdivided in three main types according to their activity: nucleosomes of neutral enhancers carry H_3_K_4_Μe_1_ histone tag, poised/bivalent enhancers are decorated with histone methylation active (H_3_K_4_Me_1_) and repressive (H_3_K_27_Μe_3_) mark at the same time, while active enhancers carry both H_3_K_4_Μe_1_ and H_3_K_27_Ac histone marks [12,13,14]. Moreover, promoters along with enhancers are the main binding sites of the Mediator Complex, which specializes gene expression patterns, recruits general transcription factors and establishes transcriptional memory between tissues and across development [15]. At a chromatin architecture level, promoter–enhancer communication is achieved through the extrusion of chromatin loops leading to the stabilization of topologically associated domains (TADs), which serve as functional genomic boundaries that restrict RE interactions and specify gene expression in a spatiotemporal manner [16]. The adjacent genomic space of TADs hosts specific motifs that facilitate binding of the CCCTC-binding factor (CTCF), a key regulator of chromatin conformation as it interacts with the cohesin complex, which acts as a chromatin loop stabilizer that dictates TAD formation [17,18,19,20,21]. Apart from chromatin architecture, enhancer transcription by itself may generate enhancer-RNAs (eRNAs- referring to non-coding RNAs transcribed from enhancer loci) [22,23]. On many occasions, eRNAs have a regulatory role in the establishment or maintenance of enhancer–promoter loops. At the level of genomic organization, enhancers can exert their regulatory function either individually or through the formation of clusters, known as super-enhancers, which concentrate transcription factor binding, are characterized by extensive eRNA transcription and serve as organizational centers for complex TAD formation [24,25,26,27].

Distinctively, all this operational heterogeneity that underlies enhancer–promoter communication ensures acute yet precise transcriptional responses at a spatiotemporal level. Given the pivotal control of regulatory elements in diverse physiological processes, such as cell-lineage specialization, differentiation, organogenesis and morphogenesis, immune cell diversification and stroma cell interactions, deregulation of their physiological function by mutations often serves as the molecular basis of pathological conditions like cancer [26,28,29]. Identification of such variants in tumor progression can be used as a diagnostic or prognostic tool to predict the clinical outcome for the patient and/or tailor therapeutic strategy in a personalized manner [30,31]. It is therefore imperative to identify and most importantly, functionally dissect genetic variability in the cancer genome that is characterized by extensive variability, in the form of SNPs, or CNAs across wider genomic areas [32,33]. International consortia document and metanalyze functional genomic experiments, providing insights regarding the effect of tumor-specific driver mutations on regulatory elements during neoplastic progression [34,35,36,37]. Such consortia are the Encyclopedia of DNA Elements (ENCODE—a public research consortium focused in identifying all functional DNA regulatory elements) [38], NONCODE which is an integrated knowledge database dedicated to non-coding RNAs (excluding tRNAs and rRNAs) [39], and The Cancer Genome Atlas (TCGA—a landmark program of cancer genomics, containing genomic, epigenomic, transcriptomic, and proteomic data from tumors, along with the clinical profile of the patients) [40].

Although some genetic variants are stably inherited and occasionally predispose for hereditary forms of cancer, carcinogenesis itself relies on inactivation of DNA repair mechanisms, leading to genomic instability and extensive accumulation of a mutational burden on a global scale [41]. Genomic variants can be further classified based on structural (SNPs or CNAs), expression (transcribed or not transcribed sequences) or functional (coding or non-coding regions) criteria, all of which ultimately reflect the mechanism through which these genetic lesions are implicated in the development and progression of human malignancies [42,43,44,45]. This review focuses on the mechanisms though which non-coding regulatory variants in transcribed or non-transcribed parts of the genome control carcinogenesis, together with the appropriate state-of-the-art methodologies to identify and study them.

## 2. Genetic Variability in the Cancer Genome

### 2.1. Structural Classification of Mutations in Cancer

**Figure 1:**
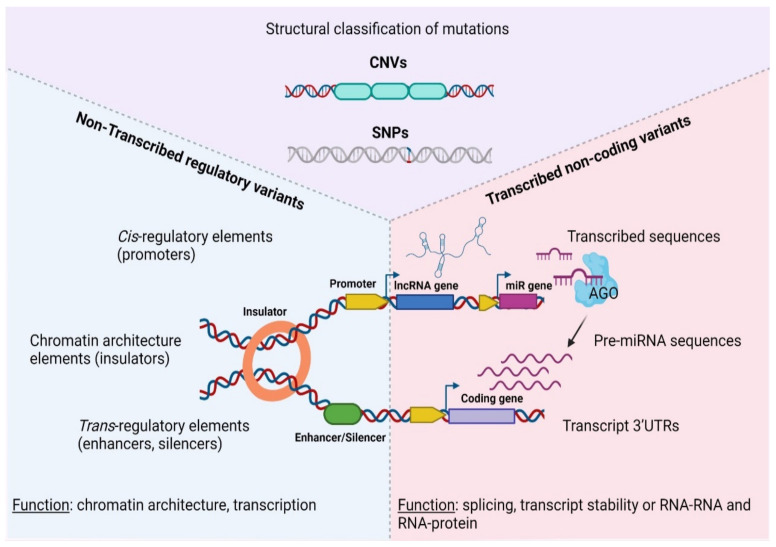
Categorization of genetic variations in the non-coding coding cancer genome. AGO—Argonaute protein, CNV—Copy Number Variation, SNP—Single Nucleotide Polymorphism, UTR—Untranslated Region, lncRNA—long non—coding RNA, pre-miRNA, precursor microRNA. Created with BioRender.com, permission: 15 July 2021.

At the molecular level, SNPs and/or CNAs can reside both in coding, as well as non-coding sequences that are either transcribed or not inside tumors (Figure 1). Depending on the nature of the mutation and the function of the underlining sequence, genetic lesions fuel carcinogenesis through a diverse array of mechanisms, including but not limited to chromatin modification, transcriptional regulation and alternative splicing, to altered transcript/protein structure or activity due to premature stop codons, non-synonymous amino-acid changes and aberrant gene fusions [46,47,48,49]. Therefore, different types of mutations or affected sequences predispose for cancer via an array of distinct mechanisms that must be examined separately.

Focusing on single nucleotide polymorphisms, genome-wide association studies (GWAS) were successful in identifying their mechanistic interplay with normal development or pathology [50]. Depending on the strength of their alleles, SNPs can be characterized as high or low risk factors for complex traits, such as cancer [51]. Nevertheless, association between SNP alleles and phenotypic impact can be confounding due to linkage disequilibrium that segregates driver mutations (that directly control the trait of interest) together with passenger mutations (that are passively inherited with the former but are regulatory neutral) within populations. Therefore GWAS approaches should be accompanied by extensive and careful mechanistic characterization with the aim of determining which of the associated SNPs are the true causative factors of the disease [52].

At the level of CNAs, two main categories can be identified: (i) germline CNAs that include duplications or deletions and (ii) somatic copy number alterations of specific loci. Germline CNAs range from 50 bp up to 1 Mb in length and predispose for hereditary types of cancer, such as familiar breast cancer. Somatic CNAs are typically longer than 1 Kbp (100 Kbp on average [53,54]) and like germline CNAs, include duplications (known as Copy-Number Gains or CNGs), or deletions (representing Copy-Number Losses or CNLs) [55]. Both types of somatic CNVs (CNGs and CNLs) are prominent lesion types in tumors that are characterized by extreme Chromosomal INstability (CIN) [56].

Presence of CNAs is heavily linked with malignant manifestation through three main mechanisms: (i) alterations in gene dosage, in which the copy number of at least one gene locus is affected [57,58], (ii) gene fusions (mainly due to genomic deletions) [59] and (iii) alterations of *cis* and/or *trans* regulatory elements [60,61,62,63]. In some cases, a correlation between copy number variations and DNA methylation status of the CpG islets for a given locus has been reported to negatively affect target gene expression [64]. Apart from cancer, germline CNAs also predispose for various developmental disorders and diseases, such as autism, schizophrenia with a parental-specific pattern of inheritance, for which they can be also potent biomarkers for prenatal diagnosis [65,66].

### 2.2. Functional Classification of Mutations in Cancer

Regardless of their structure, genomic variants (including both SNPs or CNAs) can be further classified according to their occurrence in transcribed or non-transcribed sequences (Table 1, [67]). This type of classification underpins the function through which different variants associate with disease etiology.

For example, functional variants that occur in transcribed portions of the genome generally associate with altered transcript message or function (manifested as modified exonic sequences, alternative splicing, modified UTRs, altered ncRNA folding and/or gene fusions [96,97,98]). Interestingly, most GWAS/transcribed regulatory variants are not limited to protein coding genes but primarily localize in transcribed non-coding sequences that may generate regulatory transcripts with low or no protein coding potential [99,100,101,102]. Non-coding RNAs (ncRNAs) are categorized based on their processed length, with transcripts less than 200 nt referring to short non-coding RNAs (consisting mainly of microRNAs-miRNAs [103], small nucleolar RNAs-snoRNAs [104] and piwi-interacting RNAs [105]), in contrast to long non-coding RNAs (lncRNAs), which comprise transcripts with lengths larger than 200 nt [106].

## 3. Non-Transcribed Regulatory Variants 

Variability in non-transcribed regulatory sequences (e.g., promoters, enhancers, CTCF sites) strongly associates with a mechanistic impact of non-coding variants during neoplastic development [107]. Genome-wide studies revealed an extensive correlation of these variants with conditionally deregulated spatiotemporal gene expression networks and disrupted genomic organization in various tumor forms, thus highlighting the importance of genetic non-coding variability in cancer onset and progression [67,108]. Rare SNP alleles, associated with increased risk of carcinogenesis (and/or other diseases), are enriched within expressed quantitative trait loci (eQTLs), with prominence in promoter regions of oncogenes and tumor-suppressors [109,110,111].

Apart from SNPs, somatic CNAs act as the driving force of the CIN subtype that is typical for various neoplasms. For example, 65% of gastric adenocarcinomas are categorized as CIN and since CNAs are one of the leading causes of extensive genomic and transcriptomic alterations, defining their functional role has a clinical interest [112]. Another cancer type with high percentage of CIN is colorectal cancer, in which CNVs contribute to loss of heterozygosity in *TP53* and *APC*, or amplification in *KRAS* and *FGFR1*, leading to poor prognosis due to drug resistance [113,114]. Despite of their discovery and statistical association with diagnostic or prognostic markers, such variants often lack functional characterization due to the small effect that a single SNP may have in gene expression, together with tissue-specific restrictions in the expression of the target gene [115,116,117]. Therefore, it is crucial to first stratify and subsequently present some of the elucidated mechanisms through which non transcribed regulatory variants dictate neoplastic development.

### 3.1. Genetic Variability in Promoters

**Figure 2:**
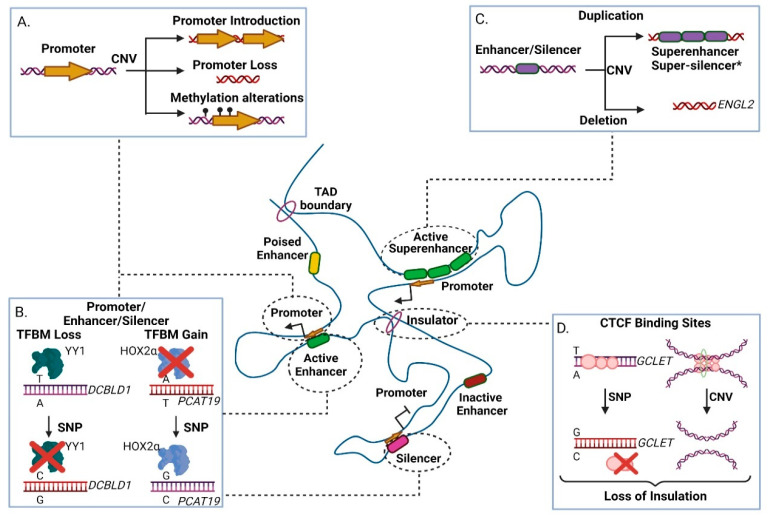
The effect of genomic variants in non-transcribed gene regulatory elements within a Topologically Associated Domain (TAD). (**A**) Effects of Copy Number Variations (CNVs) in promoters. Duplication events may lead to ectopic promoter introduction, while deletion event may result in loss of crucial promoter elements. Depending on the occasion, duplication or deletion may result in DNA methylation alterations, modulating transcriptional activity. (**B**) Effect of Single Nucleotide Polymorphisms (SNPs) in promoter, enhancer and silencer elements. Presence of SNPs may lead to either increased or decreased affinity in transcription factor binding motifs, thus altering the element’s function. (**C**) Effects of CNVs at enhancer and silencer elements. Duplications may result in creation of super-enhancers or, hypothetically*, super-silencers. Deletions may lead to loss of crucial transcription factor binding motifs, thus impairing regulatory element function. (**D**) Presence of a risk SNP to a CTCF site may lead to loss of insulation due to CTCF site disruption. CNV occurrence may also lead to loss of insulation due to deletion of a CTCF site. TFBM-Transcription Factor Binding Motif. CTCF-CCCTC-binding factor. Created with BioRender.com, permission: 15 July 2021.

There are numerous examples of genomic variants in *cis*-regulatory regions that affect transcription of coding or non-coding target genes [118,119,120]. Promoters (especially the core promoter) are the prime regulatory units of transcription, as they embed transcription factor motifs that enable formation of the Pre-Initiation Complex (PIC) adjacent to the transcription start site of the gene [121]. In many cancer types of promoter activity is altered by inherited or somatic mutations, leading to the modulation of cryptic promoter activity, loss of promoter DNA methylation or alteration (including loss or gain) of key regulatory motifs (Figure 2A, [122]).

For example, rs11672691 (G/A) and rs887391 (T/C/A), two risk-associated SNPs for poor prognosis in prostate cancer, map to a genomic region with bifunctional role acting either as promoter or enhancer. Presence of the cancer-predisposing alleles facilitates promoter-to-enhancer switching, leading to reduced binding capacity of the transcription factors NKX3.1 and YY1 to the promoter of the short isoform of the PCAT19 (Prostate Cancer Associated Transcript 19) lncRNA transcript. This favors accumulation of the long PCAT19 isoform that interacts with HNRNPAB and promotes the expression of cell cycle genes that subsequently fuel tumor growth and metastasis [68]. Another example refers to the high-risk SNP rs17079281 (C/T) that resides within the promoter of the *DCBLD1* gene and predisposes for lung cancer in Asian and European populations. The predisposing C allele of this SNP reduces the binding affinity of the YY1 transcription factor that normally represses transcription of the gene, ultimately leading to increased levels of the DCBLD1 oncogenic protein in the mutated tissues (Figure 2B) [69]. Another study in mice showed a gene directly affected by SNPs and CNAs, *Plekha5*, which normally acts as a suppressor of metastasis. In presence of SNPs or CNAs, *Plekha5* is deregulated, leading to an increase in the metastatic rate of the cells [123]. The rs2267531 SNP lies within the promoter of Glypican-3 gene (in Xq26) and the CC/C genotype of which has been correlated with susceptibility and reduced overall survival of patients with hepatocellular carcinoma (HCC) [70]. In addition, the G allele of rs2280059 SNP, which lies within the promoter of HSPH1, is able to increase its expression levels, leading to enhanced resistance of the cancer cells to treatment in patients with advanced non-small-cell lung cancer [71]. Apart from motif changes, CNVs may also alter the methylation status of oncogenic promoters (e.g., through the demise of methylation sites) leading to increased proliferation advantages for the mutated cell subpopulations, evidence of which have been extensively found in lung adenocarcinoma [124]. Collectively, genetic variability in promoter regions often associates with altered gene expression that links to disease progression of various cancer types.

### 3.2. Genetic Variability in Enhancers

Most cancer enhancers show cell- and/or stage selectivity in their activation patterns [125,126,127], therefore their associated genetic variability is ideal for assessing personalized predisposition or therapy. Since enhancers (and super-enhancers) function through DNA binding motifs, their activity is vulnerable to variation that modulates the binding capacity of transcription factor proteins, thus altering transcription of the target gene [128]. Presence of CNA (and other architectural disarrangements) combined with loss of insulation events can lead to ectopic enhancer creation or activity resulting into metaplastic differentiation associated with malignancy (Figure 2C, [129,130,131]). For example, genome-wide CNA studies have correlated a deletion in an ovarian-specific enhancer with altered expression of EGLN2, an enzyme that mediates hydroxylation and subsequent degradation of the HIF1A protein (a master regulator of oxygen homeostasis) in normoxia (Figure 2C, [75]).

Parallel to CNAs, GWAS studies have also pinpointed the association between SNPs and enhancer activity in cancer. For example, the variant rs11672691 (G/A), which resides in an intronic enhancer at the lncRNA *PCAT19* locus, correlates with prostate cancer predisposition and aggressiveness [132,133,134]. More specifically, the risk allele rs11672691-G enhances the binding activity of the novel transcription factor HOXA2, which in turn regulates expression of PCAT19 in prostate cancer through enhancer–promoter loop formation (Figure 2B) [135]. Single nucleotide editing in combination with ChIP-seq (Chromatin Immunoprecipitation followed by Sequencing) experiments revealed that binding of HOXA2 positively regulates not only *PCAT19* but also its neighboring locus *CEACAM21*. Thus, the interplay of rs11672691 with the regulatory circuit of *HOXA2*, *PCAT19* and *CEACAM21* is linked to advanced cell growth and invasion with a significant clinical impact on prostate cancer disease aggressiveness and severity, highlight the role of enhancer mutations in the regulation of neighboring coding and non-coding targets in cancer tissues [68,135].

With regards to SNPs, rs67311347 (G > A) shows a positive correlation with cancer cell proliferation in patients with Renal Cell Carcinoma (RCC). The A allele creates a binding site for ZNF8 within an enhancer element regulating the tumor-suppressor lncRNA ENTPD3-AS1, leading to its increased expression. ENTPD3-AS1 interacts with miR-155-5p and activates the expression of HIF-1a in RCC [76]. The SNP rs4693608 lies within an enhancer regulating the expression of *HPSE*, by affecting the self-regulation of the oncogenic transcription factor in acute lymphoblastic leukemia (ALL), with the A allele carriers escaping the methylation of the enhancer [77].

An independent study revealed another layer of complexity for this enhancer-like regulatory region, which seems to have a bifunctional role. The presence of additional variants that also reside in the *PCAT19* locus plays a crucial role in *PCAT19* transcript isoform generation (PCAT19-short and PCAT19-long isoforms respectively) with the PCAT19-long elevated mRNA levels determining progression of prostate cancer. Specifically, the SNPs variants rs11672691 and rs887391 that reside in the promoter region of the PCAT19-short isoform can switch the regulatory identity of the element from promoter to enhancer. Presence of these two risk alleles disturbs binding capacity of the transcription factors NKX3.1 and YY1 to the promoter of the PCAT19-short isoform. At the same time the same risk SNPs reinforce enhancer activity of the bifunctional regulatory element leading to increased expression of PCAT19-long isoform through a promoter–enhancer interaction. Subsequently PCAT19-long isoform interacts with HNRNPAB and thus influences expression of cell cycle genes leading to acceleration of tumor growth and metastasis [68].

The expression of another prostate related lncRNA, PCAT1 (Prostate Cancer Associated Transcript 1), is also modulated at the transcriptional level by a cancer-associated SNP with pivotal function in prostate cancer. Initially, PCAT1 was reported to be implicated in early prostate cancer cell proliferation, yet recently it was shown to be involved also in castration-resistant, advanced prostate tumors [84,136]. PCAT1 expression is modulated by the risk SNP variant rs7463708 (T > G) located within an enhancer regulatory element that lies 78 kb away from the PCAT1 Transcriptional Start Site (TSS). *PCAT1* promoter and its enhancer reside within a conserved TAD domain, which indicates the potential of chromatin loop extrusion between them. The T allele intensifies the binding affinity of the ONECUT and AR transcription factors, which in turn regulate PCAT1 transcription. Subsequently, the PCAT1 transcript interacts with the LSD1 and AR proteins facilitating their recruitment to enhancer regulatory elements of *GNMT* and *DHCR24* that are androgen-late response genes that correlate with prostate cancer progression [72,137,138].

Apart from prostate, SNPs in enhancers also affect other forms of cancer. rs35252396 (AC > CG) refers to a two base pair substitution variant that is strongly associated with clear cell renal cell carcinoma. This particular variant resides in an enhancer element at 8q24.21 between the genomic loci of MYC and PVT1 and along with the SNP rs6983267, whose regulatory function is well characterized in colorectal and prostate carcinoma. rs35252396 affects chromatin accessibility in this area, increasing binding of hypoxia inducible factors in this enhancer element [73,74,139,140]. rs6983267 together with rs35252396, highlight the predisposing effect of neighboring yet separately segregating regulatory genetic lesions in carcinogenesis.

### 3.3. Genetic Variability in Silencer Elements

Mutations in distal silencer elements are less understood due to the biased focus on activating enhancers, even though the latter may also act as silencers and vice versa in different tissues and cell types [141]. Silencer elements, just like enhancers, contain transcription factor binding sites, that form chromatin loops with promoters (Figure 2B,C), preferably those with high levels of trimethylation of Lysine 27 in Histone 3 (H_3_K_27_me_3_) epigenetic marker [142]. Supposedly, the formation of a super-silencer is possible, but so far there are insufficient data that support their existence [143]. A putative silencer regulating ESR1 and RMND1 expression can be found in 6q25.2, and the SNP rs910416 contained within it shows allele specific binding of MYC. This disrupts the proper function of the silencer, leading to breast cancer development [144]. Another example that highlights the function of such repressive chromatin loops, refers to the regulation of *Kit* locus by GATA1, which has a repressive role in hematopoietic differentiation [145]. Other silencers are characterized by the presence of motifs of FRA1, USF1 and USF2, EBF1, BACH2, and the RFX family among others, which display repressing activities [146,147,148,149,150]. In contrast to the binding of activators in unmethylated or lowly methylated enhancer elements, a proportion of these suppressors can bind to methylated sequences as well, indicating that some silencers may show activity even in their DNA methylated form [151,152]. The SNP rs249473 and especially the risk allele A, which lies within a silencer of the *AKT1* locus (encoding for the AKT protein, part of the PI3K/AKT/mTOR signaling pathway), abrogates its silencing activity by creating a binding site for YY1, which in turn activates *AKT1* transcription and elevates the risk of endometrial cancer [78].

### 3.4. Genetic Variability in Insulator Elements

Insulators are DNA elements which are recognized by CTCF and facilitate creation of inter-domain boundaries, conferring separation of promoters and enhancers or insulation against the spread of heterochromatin regions [153,154]. Loss of insulator elements may occur due to the presence of SNPs that alter the CTCF binding site or the methylation status of the region [155]. Moreover, CNAs that promote genomic rearrangements of CTCF sites can lead to enhancer hijacking, that associates with increased levels of a putative oncogenes, such as *MYCN* that is one of the main drivers for neuroblastoma (Figure 2D) [80,156]. The SNP rs60507107 is correlated with increased risk of lung cancer, as the A allele reduces the binding affinity of CTCF at a CTCF binding site in the first intron of *DAGLA* (in 11q12.2), leading to its altered expression in lung cancer [157]. Collectively, these examples highlight the functional diversity through which genetic variability in regulatory elements predisposes for neoplastic development and progression.

Apart from coding genes, genetic aberrations may also disturb transcriptional regulation of lncRNAs at a chromatin architecture level. For instance, GCLET (Gastric Cancer Low-Expressed Transcript) is a novel lncRNA with a gastric cancer related variant rs3850997 T > G at 16p13 in the third intron of the *GCLET* genomic locus. Expression analysis, including eQTLs, revealed a strong association between high expression levels of GCLET and improved patient survival. Moreover, in vitro experiments showed that the rs3850997-T allele is bound by the CTCF transcription factor with higher affinity compared to G allele (Figure 2D). CTCF exerts an inhibitory function, so when bound to the relevant intronic region prevents chromatin loop formation between the intron/SNP variant and GCLET promoter region, ultimately precluding lncRNA transcription [79]. Furthermore, GCLET competes with miR-27a-3p to increase FOXP2 expression, therefore affecting lymph node invasion and metastasis. Inferentially, the T allele of the rs3850997 variant represses transcription of GCLET lncRNA and absence of the transcript contributes to gastric cancer progression with a significant impact on patient clinical prognosis [79,158,159,160].

## 4. Transcribed Non-Coding Variants 

Apart from regulatory elements, cancer-related genetic variability is also embedded in transcribed, yet non-coding sequences. Transcribed non-coding Variants (referred to as TncVs thereof) exist both in coding and non-coding transcriptional units and fuel carcinogenesis through a distinct set of mechanisms compared to their counterparts in coding sequences. For example, TncVs can modulate the stability of the resulting transcript through abnormal splicing patterns, UTR variations that create or disrupt miRNA binding pockets, or through alterations in lncRNA secondary structure that influence interaction with regulatory partners (both protein and RNA molecules) [107,161]. The latter can lead to differential regulation of target gene expression, via loss of RNA-chromatin and/or RNA–protein complex formation, concurrently with disruption of TAD architecture [161]. Such cancer-related transcribed variability is not restricted to the DNA level, but also arises at the RNA level, giving rise to the very promising and largely unexplored field of epitranscriptomics which again may operate from within coding and non-coding transcripts in a similar manner to inherited mutations [162].

### 4.1. Non-Coding Variants Affecting miRNA Targeting and Biogenesis

Small RNA sequencing efforts have identified hundreds of miRNAs involved in cancer progression and tumorigenesis for a variety of cancer types and stages [163]. miRNA signatures with significant prognostic and diagnostic properties often reflect the tissue- and/or cancer-specific properties that characterize the expression of this class of non-coding transcripts [164]. In terms of function, miRNAs act on the basis of sequence complementarity with their cognate target-mRNA(s) [165,166,167]. Thus, any sequence variation, even in the form of single nucleotide polymorphisms that occurs within the seed sequence of their genomic loci, can alter targeting affinity [168]. Although GWAS approaches have revealed the importance of SNPs in oncogenic or tumor-suppressing miRNAs, functional characterization for the majority of such alterations awaits experimental validation [168,169,170].

**Figure 3:**
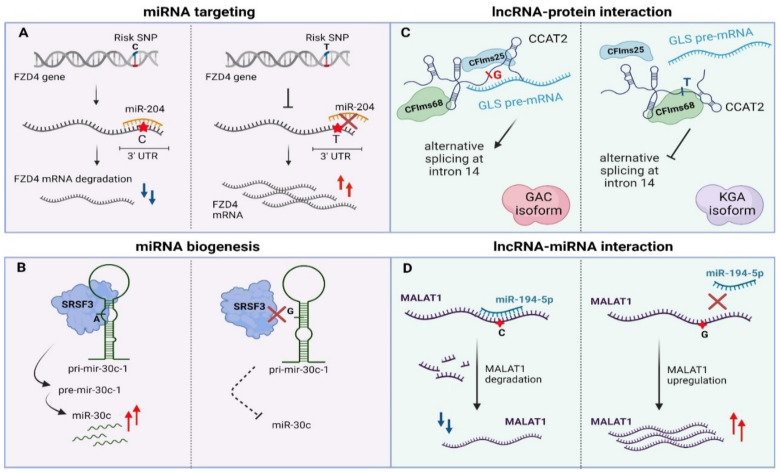
Examples of genomic variants affecting non-coding RNAs function. (**A**) 3′UTR-occurring SNPs disturb miRNA targeting via sequence-based mismatches. (**B**) SNPs in pri-miRNA transcript can alter miRNA biogenesis. (**C**) SNPs can affect lncRNA–protein interactions via alterations in lncRNA secondary structure (**D**) SNPs can alter lncRNA stability via modulation of miRNA binding sites. UTR—Untranslated region, pri-miRNA—primary miRNA, pre-miRNA—precursor-miRNA, lncRNA—long non-coding RNA, miRNA—microRNA, GLS—glutaminase, GAC—glutaminase isoform C, KGA— kidney glutaminase isoform. Created with BioRender.com, permission: 15 July 2021.

Apart from genetic lesions in miRNA transcripts, variability can also arise within miRNA binding sites in 3’UTRs of their target genes [171,172,173,174]. Such variability may ectopically create or disrupt a miRNA binding site in malignant or even pre-cancerous tissues. The miR-155-5p is highly expressed in melanoma patients and targets the 3′UTR region of TYRP1 (Tyrosinase Related Protein 1) mRNA in a SNP-dependent manner leading to decreased TYRP1 transcript levels. It has been shown that different combinations of AA/CC alleles of rs683/rs910 SNPs that lie in the 3′UTR region of TYRP1 mRNA affect the expression of TYRP1 at a post-transcriptional level while there is also a correlation with melanoma metastasis [81]. Another miR-SNP (rs713065, T to C change) in the 3′UTR region of *FZD4,* which is a consequential epidemiological biomarker for non-small-cell lung carcinoma (NSCLC), comprises a binding site for miR-204. The predisposing C allele of this SNP enhances binding of miR-204 compared to the wild type allele (T), leading to down-regulation of FZD4 through cleavage, uridylation and degradation of its mRNA. Subsequently, the miR-204-SNP mediated loss of FZD4 induces deregulation of key components of Wnt/Catenin signaling associated with impairment of colony formation and cell migration of NSCLC cancer cells (Figure 3A) [82].

An analogous example of TncV refers to SNP rs1071738 (G common allele, C minor allele in European individuals) at the miR-96/miR-182-binding site within the Palladin 3′-UTR with fundamental function in breast cancer metastasis. The ancestral C allele allows miRNA:mRNA binding while the alternate G allele disrupts it. miR-96 and miR-182 have anti-migration and anti-invasion roles in breast cancer cells that is associated with downregulation of Palladin, a phenotype which was confirmed by in vivo experiments. At the therapeutic level, in vivo delivery of miR-96 or miR-182 (fully complimentary with their binding site in Palladin-3′UTR) by using hydrogel-embedded gold nanoparticles with efficient release of miRNAs, led to a remarkable decrease of cancer cells’ metastatic capability [83]. Finally, the rs1048638 SNP that harbors within the 3′UTR of CA9 (Carbonic anhydrase IX) mRNA is strongly correlated with clinical features (overall survival, poor prognosis, recurrence) of HCC patients. The A allele of this SNP creates a binding site for miR-34a targeting that declines CA9 mRNA levels and affects cell proliferation and metastasis of HCC cells [175]. In conclusion, functional characterization of miRNA-associated TncVs in cancer progression can offer novel therapeutic opportunities at the genetic basis of cancer.

Parallel to miRNA binding sites, TncVs that reside in pri-miRNA sequences can affect cancer progression through defects in miRNA biogenesis [88,176]. A thoroughly characterized example is rs928508, referring to a G to A substitution that is present in the terminal loop of pri-mir-30c-1, perturbing its secondary RNA structure and subsequently leading to increased levels of mature miR-30c in breast cancer. Particularly, the G/A substitution, which lies in a CNNC motif, facilitates interaction of the pri-miRNA with SRSF3, a protein involved in alternative splicing and Drosha-mediated processing of pri-miRNA maturation [85,177,178]. Experimental validation with SHAPE (Selective 2’-hydroxyl acylation analyzed by primer extension, a technical approach for RNA structural analysis at single-nucleotide resolution) and toeprint assays (an assay using a fluorescent-labeled oligonucleotide to prime the reverse transcription step) proved that SRSF3 specifically recognizes the CNNC motif in a dose-dependent manner in MCF7 cancer cells [179,180]. Importantly, G/A variation promotes formation of a particular tertiary RNA structure of the pri-miRNA transcript, allowing stronger interaction with the SRSF3 protein and ultimately proper biogenesis of the miRNA (Figure 3B) [85]. Of note, the same variant has been previously linked to increased miR-30c expression in breast and gastric cancer patients [86,181]. Finally, miR-30c has also been shown to be a tumor prognostic biomarker for breast cancer, modulating chemoresistance through regulation of TWF1 and IL-11 [182].

In independent example of a mutation that affects miRNA biogenesis refers to rs7911488 (T > C), located in pre-miR-1307 and ultimately interfering with progression of colorectal cancer (CRC). Homozygous T alleles of this point mutation lead to elevated expression of mature miR-1307, which in turn binds to 3′UTR of the PRRX1 mRNA and diminishes its expression levels. Downregulation of PRRX1 enhanced proliferation and migration of CRC cells; however, the exact mechanism needs to be further clarified [87]. Finally, the A allele of rs11671784 SNP within the *miR-27a* is associated with high susceptibility to gastric cancer. Correlation studies indicated that this variant influence the maturation process of miR-27a leading to decreased expression levels of miR-27a in gastric cancer patients. The diminished levels of miR-27a activate the enhanced expression of HOXA (miR-27a-target gene) affecting tumor growth of gastric cancer cells [88]. In conclusion, genetic lesions that affect miRNA biogenesis/binding sites frequently associate with site-specific cancer progression.

### 4.2. Non-Coding Variants Affecting lncRNA Function

The last decade, lncRNAs gained particular interest in cancer biology given their cancer- and tissue-specific expression in various malignancies [161,183,184,185]. The intricate non-coding nature of their function relies on interactions with i) protein complexes (transcription factors, spliceosome, RNA binding proteins-RBPs, chromatin modifiers in the nucleus and RBPs, ribosomes and other proteins in the cytoplasm), ii) other non-coding transcripts (such as lncRNAs and miRNAs) or iii) DNA through triple helix formation [186,187]. As a result, genetic variability in lncRNA loci has also been associated with cancer predisposition, yet few cases have been experimentally validated, given the increased difficulty of functionally dissecting lncRNAs compared to miRNAs [188,189]. Among the most interesting examples of variations that occur in lncRNAs are SNPs that disorder lncRNA transcript functionality with a cancer-driving potential. Due to relaxed evolutionary constrains on primary sequence [190], lncRNA loci can easily accumulate genetic variability in cancer cells that can affect proper transcript folding, ultimately modulating lncRNA interactions with their protein partners or other regulatory molecules [191].

More specifically, lncRNAs may contribute to post-transcriptional regulation by affecting splicing [192], mRNA stability [193] or as precursors/regulators of miRNA biogenesis [194,195]. Presence of distinct sequence motifs in combination with a particular secondary structure facilitates binding of splicing factors and other RBPs enabling the lncRNA–protein functional interplay [161,196,197]. Cancer-risk variations that occur in these motifs may disturb this interaction, leading to deviant molecular signaling pathways and finally to malignant transformation [198]. An intriguing example is rs6983267 SNP (G/T) that resides in the lncRNA locus *CCAT2* (Colon Cancer Associated Transcript 2) and is correlated with colon cancer metabolism (enhanced glutaminolysis) and cell proliferation. This transcribed SNP can recruit two subunits of the cleavage factor Im complex (CFIm, CFIms25 and CFIm68 subunits) in an allele-specific manner. CCAT2 transcripts containing the G-allele, allow binding of CFIms25 with higher specificity compared to T-allele transcripts, which in turn has stronger propinquity for the CFIm68 subunit. This dominant effect regulates glutaminase (GLS) pre-mRNA alternative splicing. The interaction between *CCAT2* G-allele and CFIms25 directs binding of this RNA–protein complex to the poly(A) site in intron 14 of GLS pre-mRNA, inducing in this way splicing of glutaminase isoform C that associates with enhanced catalytic activity compared to the kidney glutaminase isoform. Biotinylated RNA pull-down experiments revealed that *CCAT2* directly binds to GLS pre-mRNA, highlighting an example of RNA-RNA–protein complex which depends on the secondary structure of a scaffold-lncRNA that is mainly affected by the rs6983267 SNP (Figure 3C, [89]).

Another example of lncRNA–protein interactions that are altered by the presence of SNP variants is lncRNA NEXN-AS1 along with its associated SNP rs114020893 at 1p31.1. This variant is correlated with increased lung cancer susceptibility and is predicted to modulate the secondary structure of the NEXN-AS1 transcript [90,199]. These examples highlight the potential of TncVs that alter lncRNA–protein interactions in disease progression, yet detailed functional insights are still required for the majority of association studies that link SNP variation with lncRNA function in cancer [188].

Apart from affecting the interplay with protein partners, there are multiple levels of lncRNA-miRNA interactions that rely on mutations with a role in cancer [200,201]. This type of mechanism is sequence-dependent, which means that any alteration in the base sequence may influence base-to-base interplay. Similar to their role in mRNA:miRNA interactions, some TncVs can affect lncRNA transcript levels through differential miRNA binding. Such an example refers to MALAT, a thoroughly characterized lncRNA in many cancer types (e.g., oral squamous cell carcinoma, melanoma) with a predominant oncogenic activity, although a tumor-suppressive function has also been reported in breast cancer [202,203,204,205]. The first functionally characterized SNP of MALAT1 was rs664589, which is involved in colorectal cancer progression via its interaction with the miR-194-5p. MiR-194-5p targets MALAT1 for degradation in a rs664589 allele-dependent manner. Binding of miR-194-5p to the MALAT1 transcript with the rs664589-C genotype targets it for degradation in the nucleus, in contrast to the G allele that decreases overall binding affinity of the miRNA, leading to accumulation of MALAT1 and ultimately poor patient survival, increased distant metastasis and enhanced tumor growth (Figure 3D, [91]).

Another example of non-coding variant that affects miRNA-lncRNA interplay in cancer is CCSlnc362. CCSlnc362 (RP11-362K14.5) is a recently identified tumor-promoting lncRNA in colorectal cancer. Its expression correlates with the SNP variant rs1317082 (T > C), located at exon 1 of the CCSlnc362 locus. Functional experiments linked the oncogenic role of the CCSlnc362 with acceleration of the cell cycle parallel to apoptotic blockage. In vitro luciferase assays showed that miR-4658 binds to CCSlnc362 in an allele-specific manner. Binding affinity of miR-4658 is increased in the presence of homozygous C alleles in contrast to the T allele, highlighting an allele-dependent predilection of the miR-4658 seed [92]. Finally, a correlation study of rs11752942 (A > G) SNP located in LINC00951 (lincRNA-uc003opf.1) exon, conducted in 1493 Esophageal Squamous Cell carcinoma (ESCC) patients, revealed a distinct association of the G risk-allele with the reduced expression of LINC00951. The regulation of LINC00951 is miRNA-149 mediated and is involved in ESCC cell proliferation and tumor growth [93].

An independent study focused on the long intergenic noncoding RNA (lincRNA) LINC00673, which is correlated with an antitumor effect in pancreatic ductal adenocarcinoma. Rescue experiments divulged the significant role of this transcript in cell proliferation mechanism of pancreatic cancer cells while in vivo xenografts experiments showed its implication in pancreatic cancer tumor growth. LINC00673 promotes PTPN11 ubiquitination and degradation via mediation of an PRPF19–PTPN11 interaction, resulting into an elevated and STAT-dependent anti-tumor response. The function of LINC00673 was strongly linked to the germline variant rs11655237 (G > A transition), which creates a binding site for the miR-1231 preventing LINC00673 from exerting its regulatory role. Similar to the previous examples, miR-1231 acts with preference to the A allele of the rs11655237 variant, serving as a decoy for LINC00673 function [94]. Another study of lncRNA SNPs variants in a cohort of 505 nasopharyngeal carcinoma patients, uncovered variants associated with chemoradiotherapy sensitivity of patients. Specifically, *MEG3* rs10132552 CC genotype was linked to elevated toxicity, *LINC-PINT* rs1059698 CC had a protective role against neutropenia and myelosuppression and *pR-lncRNA-1* rs73594404 GA genotype patients had increased risk of toxic reactions. All the mentioned lncRNAs are involved in p53 signaling network, a fact that highlights their SNP potential for reducing treatment toxicity [206].

In the same context of ncRNA interactions, some polymorphisms indirectly modulate isoform selection [207]. An example of a miRNA:lncRNA interaction that relies on transcribed SNPs and associates with isoform stabilization of a Receptor tyrosine kinase (RTKs) target in cancer, is EGFR-AS1 (EGFR Antisense RNA 1). RTKs are of great importance in cancer progression with pivotal clinical and therapeutical applications [208,209,210,211,212,213,214]. rs10251977, which associates with the lncRNA EGFR-AS1, normally stabilizes isoform A of its RTK target EGFR in oral cancer patients. EGFR-AS1 was suggested to act as a scaffold for PTBP1 (member of the heterogeneous nuclear ribonucleoprotein family) to promote EGFR-A stabilization. The minor allele A of rs10251977 creates a binding site for miR-891b (which is downregulated in tumors), leading to degradation of EGFR-AS1 and thus is correlated with elevated levels of the alternative D isoform of the EGFR [95]. Although this study needs further experimental validation, it represents a notable example of a natural antisense transcript that regulates its mRNA target *in cis* through a genetic variant. This type of genetic variation that alters isoform selection of well-defined oncogenic drivers like EGFR may expand the prognostic toolbox of cancer or meliorate the personalized therapy for the patient. Genetic variation could also affect ectopic biogenesis of miRNAs from lncRNA loci cancer, however experimental validation of such cases in still pending.

## 5. Methodologies to Functionally Characterize Non-Coding Variants

There are many bioinformatic strategies and databases that take advantage of cancer genomic data to conduct significant correlations of cancer risk and predisposition (Table 2).

However, validation and most importantly functional dissection of cancer-driver and passenger mutation requires innovative experimental approaches [215,216,217,218,219]. Alongside the advancement of genomic techniques and next generation sequencing technologies that improved our understanding regarding the function of the genome, came pioneer research strategies that identify, validate and finally characterize non-coding variability.

Discovery of functional variants in the non-transcribed portion of the genome is inextricably bound to experimental approaches designed to unveil novel regulatory sequences. A hallmark of a regulatory sequence is chromatin accessibility that subsequently allows functional activation of the region through binding of transcription factors [220]. Therefore, general approaches that scan the genome for open chromatin can serve as the first step towards the identification of regulatory variants, especially in *cis* regulatory elements [221,222,223]. When it comes to *trans* regulation (enhancers, silencers, insulators), chromatin status needs to be complemented with experimental assessment of chromatin architecture in the cancer genome in order to pinpoint the target(s) of the regulatory sequence [224]. Most importantly, the causative motif(s) within these *cis* or *trans* regulatory sequences needs identification and experimental validation prior to any connection with genetic lesions. Below some of these experimental approaches are presented based on the function and position of the non-coding regulatory sequence that hosts the causative variant with regards to it target(s).

### 5.1. Scanning for Regulatory Sequences Based on Open-Chromatin State

DNase was first used during the 1980s to map regulatory elements through the identification of global chromatin accessibility [225]. Following nuclei isolation, permeabilization of the nuclear membrane and DNaseI incubation, DNA elements are enriched by size selection either with gel extraction or by ultracentrifuge purification. The methodology can then be coupled with next generation sequencing. The result of this process is the detection of DNaseI Hypersensitivity Sites (DHS), which are sites located within open-chromatin regions with median length of 300 bp that are protected from degradation by DNaseI due to presence of transcription factors (TF) (Table 2) [226]. DNase-seq has the advantage of detecting open-chromatin without requiring prior knowledge of the sequence of the TF bound to the DHS. Additionally, it has higher sensitivity than other approaches (see below) at promoters. Its drawbacks are linked to the sequence specific function of DNaseI, thus DHS global identification is not bias-free. Moreover, the purification steps may lead to loss of DNA sample, lowering overall sensitivity of detection [227,228,229,230]. Although DNase-seq vastly enriches the sample in promoter regions, it demonstrates decreased representation of regulatory elements in a condensed chromatin state. Such an example applies to cases of SNPs within promoters of imprinted genes, where the methodology would show allelic biases towards the imprinted allele [228].

Variants detected within DHS are strong candidates for having a key role in carcinogenesis. Such variants are the SNPs rs62331150 (within active promoter region) and rs73838678 (within strong enhancer region), which are correlated with increased risk for breast cancer [231]. Complementary to SNPs, presence of CNVs in DHS may result in large scale chromatin accessibility changes. Such an example is deletion of the DHS chr8:579137-581436, which leads to increased expression levels through enhanced promoter accessibility of several tumorigenic protein-coding and non-coding genes [232].

An improvement of DNase-seq refers to single-cell DNase-seq (scDNase-seq). Application of scDNase-seq enables study of gene promoters and enhancers at a single-cell level with highly reproducible results. One SNP identified with this methodology is the chr18:52417839 (G > C), the frequency of which increases in patients with thyroid carcinoma, leading to decreased expression of TXNL1, due to disruption of a p53 binding motif within its promoter [233].

Formaldehyde-Assisted Isolation of Regulatory Elements sequencing (FAIRE-seq) can be used independently or in combination with other genomic approaches for the discovery of accessible chromatin, as it enriches for nucleosome-depleted DNA [234]. Following a cross-linking step, the non-crosslinked and therefore accessible DNA that may host interactions with TFs is isolated. Coupled to next generation sequencing, this method serves as a first, yet often crude, option for identifying accessible chromatin. The simplicity of the method together with the fact that it does not require prior treatment of the sample, highlight its applicability (Table 2). In comparison with DNase-seq, it shows reduced bias for *cis* regulatory regions and greater sensitivity in detecting intronic and intragenic regions. Its downsides associate with low signal-to-noise ratio, demanding high fixation efficiency, making data analysis more difficult, while it does not provide direct functional clues and therefore demands coupling to other techniques [234]. Micrococcal nuclease digestion with deep sequencing (MNase-seq) utilizes a non-specific endo-exonuclease micrococcal nuclease to detect chromatin regions bound by proteins [235,236]. MNase-seq requires low sequencing depths [237] but relies on non-specific digestion [238].

Proposed as an improved strategy for identifying candidate regulatory elements [239], the Assay for Transposase-Accessible Chromatin with high-throughput sequencing (ATAC-seq) is currently the most efficient method of this category. It utilizes the use of a hyperactive Tn5 transposase (normally catalyzes the movement of a transposon to accessible chromatin) that adds deep sequencing adaptors to accessible genomic regions. The superiority of this method is linked to its significantly increased efficiency, allowing for reduced cell numbers as input sample while the duration of the protocol is relatively short (Table 2). Additionally, it enables interrogation of both nucleosome and TF occupancy in regulatory elements. Like FAIRE-seq though, it does not provide direct evidence for the function of the regulatory element, thus needs to be coupled to other methodologies tailored for functional dissection [237,239]. So far, it has been successfully used along with eQTL analysis to detect variants correlated with altered chromatin accessibility for schizophrenia [240].

### 5.2. Scanning for Regulatory Variants in Trans Regulatory Elements

Massively Parallel Reporter assays (MPRAs) [241] and CRE-seq [242] have been extensively used for enhancer mapping, by creating a library in which the candidate enhancer sequences are inserted upstream of a reporter gene, regulating its expression.

Apart from insertion of the enhancer sequence in the plasmid, an extra step for barcode insertion in the 3′UTR is required, to pinpoint single enhancer mapping. This barcoded library is then transfected to cells that are harvested after 24 h, optionally followed by sorting and deep sequencing of the positive clones. As a result, the normalized count of the individual barcodes can be used for the estimation of regulatory activity of each enhancer in a particular cell line. This method allows high-throughput examination of enhancer activity, as well as multiple independent examinations of each enhancer, by use of different barcodes (Table 2). As for its drawbacks, it is an episomal assay, thus the enhancer activity is measured outside its native context, while insertion of the candidate sequences upstream of the TSS of the reporter gene may add false positive biases for enhancer activity in cases that an inserted sequence acts as a promoter instead of enhancer [243,244,245,246].

Self-transcribing active regulatory region sequencing (STARR-seq) is a high-throughput methodology to study enhancer activity [247]. With this approach, candidate enhancer sequences from various samples may be tested as input. These putative enhancer elements can be sonicated fragments previously enriched in functional elements by a FAIRE step, selected Bacterial Artificial Chromosomes (to study parts of larger genomes, including the human genome) or even synthesized oligonucleotides (which are used for studying the role of SNPs and SNPs in enhancer function). STARR-seq exploits the ability of the enhancer to act independently of its position, as the putative enhancer sequence is inserted in the 3′UTR of a reporter gene (e.g., luciferase, GFP) that lies within a plasmid used to transfect cells in culture (Table 2). Since the candidate sequences lie within the transcript area, barcoding is not required to perform this analysis thus making library construction easier [129]. A single day post transfection, positive cells are sorted and subjected deep sequencing, to identify which candidate sequences indeed act as enhancers. Enrichment of each candidate enhancer in the cDNA sample provides quantitative data for the activity of each enhancer [248]. This offers the ability to parallelly distinguish the activity of the same enhancer in presence of different variant alleles [249,250]. STARR-seq drawbacks relate again with the episomal nature of the assay, the fact that sequence insertion within the transcript may affect its stability and thus the result of the measurement and finally the fact that the assay does not allow multi-vectoral examination of the enhancer’s activity [251]. Applications of this methodology extend beyond studying cell-type specific enhancer activity. It can be applied for studies focusing on finding enhancers with a hormone or drug response potential or for comparative genomic approaches [13,252,253]. One advantage that distinguishes STARR-seq from similar approaches is the use of candidate silencer regions, in which the drop in the reporter gene expression levels can been effectively used for silencer element characterization [141].

### 5.3. Scanning for Regulatory Variants Based on Chromatin Interactions

Identification of TREs (enhancers, silencers and insulators) presents technical challenges that relate with the distance and orientation of the regulatory element compared to target CREs. Strategies such as Hi-C interrogate chromatin architecture through a combination of proximity ligation and deep sequencing [254]. However, such general approaches can be misleading since not all chromatin interactions are functionally relevant. Approaches that combine Chromosome Conformation Capture methodologies with a Chromatin Immunoprecipitation (ChIP) approach, such as Chromatin interaction analysis with paired-end tag sequencing (ChIA-PET), HiChIP and Proximity Ligation-Assisted ChIP-sequencing (PLAC-seq), isolate the fraction of chromatin interactions that are functionally relevant because they coincide with binding of transcription factors, CTCF or RNA-Pol-II [255,256].

The ChIA-PET strategy relies on crosslinking that fixes nuclear architecture, followed by chromatin extraction and sonication. Subsequently, an antibody is used to enrich for chromatin loops that contain the interacting protein of interest (e.g., RNA Pol-II). After enrichment, a linker ligation step is performed, in which the sample is separated in two aliquots and a different half-linker oligonucleotide is added in each sample fraction. Then both aliquots are mixed once again to perform the proximity ligation assay of the half-linkers that interact with each other. Ultimately DNA fragments are extracted after a de-crosslinking step, followed by protein digestion with *Mme*I to create paired-end tags with a tag-linker-tag order. Sequencing of the sample, most prominently by utilization of the Illumina platform, followed by a complex bioinformatic analysis can reveal the fraction of chromatin interactions that involve the protein of interest [257]. Using transcription factors as baits, ChIA-PET has the advantage of recovering global maps of precise interaction points on a genome wide scale, enabling the study of SNPs that lie within DNA regulatory domains, making it a great tool to study variants in promoters, enhancers, silencers and insulator elements at once (Table 2). Its pitfalls mainly associate with its complexity both in terms of sample preparation and bioinformatic analysis, together with its inefficiency that warrants extreme sequencing depths and therefore significantly elevated cost [258]. Moreover, it does not provide any information with regards to actual transcription, thus coupling its results to an RNA-targeting approach is required [259]. Nevertheless, ChIA-PET has been utilized for proving chromatin looping that is mediated by estrogen receptor alpha in hormonal cancer [260], or for characterization of CTCF-mediated loop formation [261]. Development of long-read ChIA-PET (250 bp tags) has improved mapping efficiency, enabling the study of SNPs and/or haplotype-specific interactions in various cancer types, most prominently breast cancer [262,263]. Such an example is rs16904316 that lies within the gene-body of the enhancer-like lincRNA CCDC26. Its promoter overlaps with a super-enhancer and shows correlation with onset of ALL in children. ChIA-PET analysis showed a cell-type specific long-range interaction between CCDC26 and MYC, indicating the regulatory role of lincRNA loci in MYC overexpression in ALL [264].

HiChIP is a protein-centric chromatin conformation capture method, which is based on the principles of in situ Hi-C and transposase-mediated on-bead library construction [265]. The protocol relies on cross-linking in vivo, followed by nuclei isolation and in situ Hi-C contact generation. Following establishment of long-range DNA contacts, nuclei lysis and sonication of the sample is performed prior to ChIP. Hi-C contacts carrying the target protein are then used for on-bead Tn5 library generation for paired-end sequencing. In comparison to ChIA-PET, it shows higher efficiency (10-fold increase in the yield of conformation informative reads) and lower false-positive ratio (Table 2). Furthermore, HiChIP requires up to 100-fold less starting cell amount than ChIA-PET, while also having a 2-day span workload [265]. A computational method to analyze data derived from HiChIP experiments is FitHiChIP, which can be applied to compute statistical significance estimates, lower the background, and overcome possible biases of the method [266]. HiChIP has been utilized to detect SNPs within active chromatin regions (carrying H3K27ac labeling) interacting with *TNFAIP3* promoter. Some of the SNPs detected by this methodology showed allele-specific expression profiles (such as rs538522, rs559766217) and allele-specific TF binding profile (the rs643177 for TF Pou2f1) [267]. Another study utilized HiChIP to detect promoter–enhancer loops within risk-associated loci for endometrial cancer. The results of this analysis found four high-risk correlative variants. Among those variants were rs882380, which regulates the oncogene *SNX11* and tumor suppressor *HOXB2* and rs937213 that regulates the oncogene *SRP14* (prognostic marker in renal cancer). Additionally, SNP rs7579014 that regulates the context-dependent tumor suppressor *BCL11A,* was proven to be a high-risk correlative variant in endometrial tumor sample. Finally, SNP rs9600103 was found to be part of a 23 bp anchor looped to the promoter of *KLF5* [268].

PLAC-seq has a similar philosophy to ChIA-PET, differing in the proximity ligation step that occurs in nuclei, prior to lysis and sonication of the chromatin [269]. Thusly, the efficiency and accuracy in detecting long-range chromatin interactions via PLAC-seq is again vastly increased compared to ChIA-PET (Table 2). The cells required for a PLAC-seq protocol are up to 200-fold less than those required for ChIA-PET. Additionally, it produces less inter-chromosomal pairs and more intra-chromosomal pairs than ChIA-PET, covering more regulatory elements (supported also by DHSs) [269]. So far, PLAC-seq is used along with other methodologies such as ATAC-seq, to identify putative functional SNPs that show correlation with Alzheimer’s disease (AD). This approach pinpointed SNP rs10130373 as a single variant with an important functional role in AD, along with various other SNPs like rs181391313, which causes a KLF4 site disruption in a putative microglia-specific intronic regulatory element in *STAB1* [270].

### 5.4. Scanning for Regulatory Variants Based on RNA-Chromatin Interactions

In the metagenomic era, regulation of the genome largely depends on direct or indirect interactions between the chromatin of regulatory elements and lncRNA transcripts. Such RNA-chromatin interactions are crucial for the recruitment of histone writers [26,271], interaction with transcription factors [272,273], formation of triple helixes or R-loops with DNA [274,275] and/or maintenance of chromatin loops [276]. Chromatin Isolation by RNA Purification sequencing (ChIRP-seq) is among the most utilized methodologies to study RNA-chromatin interactions [277]. This approach again relies on cross-linking for stabilization of RNA-chromatin interactions, followed by capturing of target transcript with multiple 20-mer antisense DNA probes that are biotinylated. After elution, the enriched DNA fragments are deep-sequenced, enabling discovery of putative regulatory RNA binding sites in the genome. Among the pitfalls of ChIRP-seq are low expression issues of the endogenous RNA target that can lead to an increased number of false-positive hits by the capturing probes (Table 2). ChIRP-seq has been successfully used to identify SNPs in various DNA regulatory elements, with an example referring to the detection of polymorphisms related with prostate cancer progression in lncRNA regulatory elements, such as rs72725879 and rs7463708 SNPs which lie within an enhancer element of *PCAT1* [72].

Apart from ChIRP-seq, similar methodologies referring to Capture Hybridization Analysis of RNA Targets sequencing (CHART-seq), Mapping RNA-Genome Interactions (MARGI) and in situ MARGI (iMARGI), Global RNA Interactions with DNA by Deep Sequencing (GRID-seq), Chromatin Associated RNA sequencing (ChAR-seq) and RNA And DNA Interacting Complexes Ligated and sequenced (RADICL-seq) have also been used to clarify the role of SNPs in RNA-chromatin interactions [278]. CHART-seq incorporates an extra step of RNase H sensitivity assay to further improve specificity compared to ChIRP-seq, while also provides larger fragments for the analysis, albeit with lower sensitivity [279,280]. MARGI and its upgraded version iMARGI) have also been applied in similar approaches due to minimization of potential sequence biases given the increased read lengths that they offer [281]. These protocols are more straightforward but require millions of cells and comes with a lengthy workload. Additionally, difficulties in sensitivity and specificity evaluation do exist, without a clear statistical model being optimal for downstream bioinformatic processing [282].

GRID-seq has more specificity for binding to RNA or DNA, with low background noise along with an established computational pipeline for detecting background RNA-DNA interactions [283]. GRID-seq detects multiple RNA classes on chromatin, both acting in *cis* and in *trans*, while it also provides information on interactions in the 3D genome as it showcases formation of enhancer–promoter loops [283]. As for its limitations it requires extensive deep sequencing for creating the contact maps, otherwise RNAs with low abundance may not be detected, while it poses mapping issues in low complexity regions [284]. Alternative approaches include ChAR-seq that offers increased technical specificity and improved resolution in comparison to MARGI, while in comparison to GRID-seq, it has lower chance of false mapping [285]. Finally, RADICL-seq is the most recent method for studying genome wide RNA-chromatin interactions, allowing study of 3D nuclear structures. It offers four advantages in comparison to other methodologies that can be summarized to greater resolution, decreased background noise, reduced fraction of nascent RNA-chromatin interactions and improvement of fragment selection as well as downstream alignment steps. Overall, it offers an improved performance/cost ratio than GRID-seq [64,196].

### 5.5. Methodologies to Functionally Dissect Transcribed Non-Coding Variants

Given their transcribed nature and radically different mechanism of function, TncVs require a radically different set of experimental strategies compared to their non-transcribed regulatory counterparts. RNA Antisense Purification Sequencing (RAP-seq) is a methodology applied to the characterization of TncVs in the context of ncRNA secondary structure and interaction [286]. Based on the cross-linking of macromolecular complexes formed by ncRNAs alongside with the use of antisense biotinylated oligos, RAP-seq enables capture of ribonucleoprotein complexes and thus identifies proteins and/or RNA that interact with the target RNA on a genome-wide scale (Table 3).

Its main disadvantage is background noise that is significantly alleviated through stringent hybridization and/or wash conditions [286,287]. RAP-seq has been effectively used along with other tools to identify the SNP rs199971565 as a novel indel biomarker for gastric cancer susceptibility, as it affects the secondary structure formation of miR-302c [288].

Another promising methodology for studying RNA–protein interactions is RNP network analysis by mutational profiling (RNP-MaP) [289]. Use of the cell-permeable reagent NHS-diazirine (SDA) allows rapid labeling of RNA molecules at in live cells. Activation of SDA by UV leads to formation of bonds between lysine residues in proteins with ribose base moieties at a 4–9 Å distance. After crosslinking lysis is performed, followed by protein digestion, leaving short peptide adducts. Utilization of the MaP reverse transcriptase, that reads through the adducts with relaxed fidelity, creates mutations at the RNA–protein interacting sites. RNP-MaP can be applied both for single-stranded and double stranded parts of the RNA molecule, showing a slight preference for single-stranded molecules (Table 3). Bond formation on the RNA molecule is independent of the nucleotide(s) found in the binding site, although it has higher reactivity with adenosine and uridine. RNP-MaP can be efficiently coupled with mass spectrometry approaches for identifying the interacting proteins. Thusly, RNA-MaP can be effectively used to identify the critical regions for protein interaction in ncRNAs and mRNA, and how these networks form and dissociate in different cell-types in presence of variants [289].

Another in vivo methodology for detecting RNA–protein interactions that relies on the Clustered Regularly Interspersed Palindromic Repeats (CRISPR) technology, is CRISPR-Assisted RNA–protein Interaction Detection (CARPID) [290]. CARPID utilizes a nuclease-activity-free form of the RNA-targeting VI-D CRISPR single effector dCasRx system, fused with the BASU biotin ligase. The dCasRx effector is capable of processing two sgRNAs simultaneously, thus increasing specificity. The BASU biotin ligase adds biotin groups to proteins interacting with the target lncRNAs, ultimately facilitating immunoprecipitation using streptavidin-bound beads and identification by mass spectrometry (Table 3). Coupled to RNA-seq, CARPID determine allelic expression and formation of RNA–protein interactions, based on the availability of SNPs and indels in the genetic background [290].

Post-Transcriptional Regulatory Element sequencing (PTRE-seq) is a high-throughput massively parallel methodology to study the effect of 3′ UTR sequence in post-transcriptional regulation via miRNA targeting [291]. It utilizes plasmid vectors that carry a reporter gene, with an insertion site for the candidate regulatory element (along with a unique barcode) in the 3′ end of the reporter. These vectors are then used for library construction of all candidates 3′ UTR regulatory elements followed by cell transfection. RNA-seq can be used to estimate barcode counts for each regulatory element (Table 3). The fact that PTRE-seq allows use of synthetic 3′ UTR sequences as inserts, may allow future applications aiming towards the characterization of TncVs in this regulatory context [291].

Parallel analysis of RNA-structure sequencing (PARS-seq) is a methodology that provides information about the secondary and tertiary structures of RNA molecules [292]. The principle of the methodology is based on the digestion of the RNA molecules with RNases that are specific for single-stranded and double-stranded RNA. The resulting fragments are reverse-transcribed to cDNA and deep-sequenced. This results in high-resolution sequences of the RNA, that can be used to deduce the RNA structure based on the comparison of the different digestion patterns by the various RNases (Table 3). PARS-seq has the advantage of providing RNA structural information that distinguishes between paired and unpaired bases. As for its disadvantages, use of RNases might not be specific, while digestion conditions need to be very well optimized, as the RNA can be over-digested. Finally, this application can only be performed in vitro [293]. Variants that may affect an RNA secondary structure might be detected by this approach, although there not any examples yet [294].

### 5.6. Validating Regulatory Variants with CRISPR-Based Approaches

Many applications of the CRISPR associated protein (Cas) system have been used to validate regulatory variants. Classical CRISPR-Cas9 editing can be deployed for altering the DNA of regulatory sequences in which a variant occurs (mostly tailored to SNPs but can be applied to CNAs in some cases) (Table 4). The editing strategy can rely either on correcting the cancer predisposing allele or creating it in an otherwise non-cancerous background. Mutated versions of Cas9 with nickase activity combined to the use of two distinct sgRNAs for a single target, which effectively reduces off-target effects are the preferred strategy for precise SNP editing [295,296].

CRISPR-activation (CRISPR-a) [297,298] or CRISPR-inhibition (CRISPR-i) [299], both of which rely on catalytically inactive forms of Cas9 that are coupled to transcriptional activators or inhibitors to endogenously modulate transcriptional activity, can precede SNP editing in order to precisely link the surrounding sequence of the variant with gene expression of specific targets [300,301]. Examples include a CRISPR-a based methodology that has been applied in the study of mutations in regulatory elements of *KRAS* in colorectal cancer [302].

A CRISPR-i approach has been used in the study of SNP rs11986220 (A > T), which resides in an enhancer that regulates expression of *MYC* and *PVT1*. This study correlated this SNP with increased DNA methylation levels at a nearby CTCF site that lies between the enhancer and the promoter of MYC and PVT1, leading to loss of insulation and formation of an enhancer–promoter loop which increases the expression level of MYC and PVT1 in prostate cancer (the same occurs also in presence of CNA that destroys the CTCF binding site) [303]. Application of the CRISPR armament can be expanded with the use of Cas9 homologs from species other than *Streptococcus pyogenes*, which recognize different PAM sequences allowing the study of loci that are not decorated with classical PAM sequences, or other Cas proteins like Cas12 and Cas13 that can be used for increased specificity and RNA targeting, respectively [304,305,306].

A CRISPR-deletion based approach was used to study the effect of two prostate cancer risk-associated SNPs, rs12144978 and rs4919742, involved in loop formation of two distinct prostate cancer risk-associated CTCF sites. The two CTCF sites regulate the expression of *KCNN3* and *KRT78*, by insulating the promoters of each gene from active enhancer. Deletion of these CTCF sites leads to ~100-fold increase in the expression levels of each gene due to enhancer adoption [307]. Another example of a CRISPR-based approach for the functional characterization of an SNP focused on rs2431697. This SNP lies within a cell-type specific enhancer that forms a cognate enhancer–promoter loop with the promoter of miR-146a, a miRNA with significantly downregulated expression in patients with Systemic lupus erythematosus. Presence of the high-risk T allele lowers the binding affinity of NF-κB binding site, which then leads to the decreased expression levels of miR-146a (this result was also verified by FAIRE-seq and ATAC-seq) [300].

## 6. Utilizing Non-Coding Variants in Clinomics

Clinical interpretation of genomic variants in cancer relies on a multidimensional methodology, which incorporates an array of computational algorithms and bioinformatic approaches coupled to large scale genomic data of cancer patient cohorts from different types of cancer [308]. So far focus has been given on the identification of somatic mutations in coding sequences with cancer-driving potential together with their clinical pertinence, reflecting the wealth of exome sequencing data that is currently available at an improved depth/cost ratio compared to whole genome sequencing [309]. In addition, annotation of human protein coding genes is well defined and together with availability of amino acid sequences, allows precise evaluation of mutational events on protein function [310].

More recently, whole-genome sequencing data have shed light into the non-coding part of the genome and comprehensive studies focusing on non-coding variants [311]. A continuously growing list of genome-wide studies that focus on non-coding variants, reveal their crucial potential in cancer clinomics and diagnostics (Table 5, [312,313,314]). Robust statistical algorithms are now available to detect rare genomic variants with prognostic potential, a methodology which leads to the development of precision therapeutics [110]. Nonetheless, comprehensive studies focused on the clinical utilization of non-coding variants in cancer are limited. A recent pan-cancer analysis of solid tumors revealed 21.574 recurrent non-coding mutations in patient genomes, 580 of which were cancer related when correlated with TCGA clinical features [315]. This study emphasized on mutations that occur in TF binding sites through analysis of epigenomic and chromatin structural data, divulging TEAD1- CX3CR1- and NFYB-bound enhancer elements as the top-scored regulatory sites with high mutational significance in cancer.

Another recent study focused on the correlation of 3′UTR variants with clinicopathological features of breast cancer patients as well as with chemotherapy response. The investigated variants included the *DPYD*-associated rs291593-CC, which is correlated with increased risk of toxicity in cancer patients receiving 5-fluorouracil chemotherapy and the *AKR1C3*-associated rs3209896-AG, which is linked to elevated risk for breast cancer recurrence. Moreover, the progesterone receptor-associated rs1824125-GG was found to be associated with shortening of progression-free survival, while the *ALDH5A1*-associated rs1054899-AG/AA correlated with reduced chemotherapeutic response to 5-fluorouracil, doxorubicin and cyclophosphamide [316,320,321]. Survival analysis indicated shortening of survival time in patients carrying the rs7756222-CC and rs9487402-TG/GG variations in *SLC22A16* gene [316,322]. These data demonstrate the potent existence of clinically associated variations in progesterone signaling pathways.

Ancestry across tissue and cancer types has been interconnected with the molecular and genetic background of African, European, South Asian and East Asian cancer patients [36]. Despite limitations in sample size (only 17% non-European samples), this approach revealed a significant correlation of ancestry with miRNA variations with greater differences in individual cancer types when compared to pan-cancer analysis. Approximately 80% of ancestry-associated miRs were located within host genes, indicating the need of considering (epi)genetic factors in the association of miRs with ancestry. This pilot study reflects the need of considering ancestry lineage in genomic variant research, especially for non-coding variants which may present a distinct alteration pattern among lineages.

A similar study of genetic variation in 1524 miRNA genes aimed to investigate the distribution of variation in diverse human populations including European, Asian and African populations (69 unrelated individuals in total among 14 global populations). Intriguingly, novel pre-miRNA hairpin mutations, located in highly conserved miRNA seed genomic loci with different frequencies among the individuals, were identified. Linked to cancer, the T allele of SNP rs12355840 in hsa-mir-202, previously reported to interfere in breast cancer mortality, had a significantly higher frequency in non-African populations (65%) when compared to African ones (26%) [323]. The same T-allele was also linked with a reduced risk of breast cancer mortality but was related to Hodgkin lymphoma [324,325,326].

A significant correlation was performed between 505 colorectal cancer (CRC) patients’ clinical profile and the SNP variant rs13230517 that lies in the promoter region of RP11-3N2.1 lncRNA. RP11-3N2.1 was shown to be downregulated in colon cancer biopsies and patients with rs13230517 GA/AA genotype had lower risk of developing colorectal cancer compared to AT/TT genotype [317]. In the context of the same cancer type, another study was conducted based on 900 CRC patient biopsies focusing on rs531564, C > G in the pri-miR-124. Although the exact molecular function of this polymorphism is currently unknown, a detrimental connection to clinicopathological features was achieved, revealing an association of rs531564 with lymph node metastasis and poor differentiation [317].

An independent association study of 359 HCC patient clinical characteristics with non-coding variants emphasizes in gene polymorphisms of *H19* lncRNA. This study divulged a novel SNP variation (rs3741219) in the fifth exon of the transcript that is linked to elevated risk of developing HCC [318]. Additionally, a meta-analysis based on 11,821 HCC patients in total, shed light into miRNA SNPs serving as biomarkers for HCC. Specifically, the hsa-mir-146a residing rs2910164 (G to C variation) was linked to reduced HCC risk for the C allele while hsa-mir-34b/c rs4938723 (T to C variation) was associated with increased HCC risk. Furthermore, hsa-mir-196a-2 rs11614913 and hsa-mir-149 rs2292832 variations were all correlated with elevated HCC risk [319].

A pan-cancer study focused on enhancers of approximately 9000 samples in 33 different cancer types, highlighted the positive correlation of overall enhancer activation with aneuploidy in cancer. FANTOM Project (annotated enhancers based on epigenetic marks, TFs binding and open chromatin state) and TCGA data were utilized for this systematic analysis [327]. More specifically, tumor enhancers were divided into three subgroups based on their active state and then each group was interrelated with single CNAs and point mutations. Interestingly, highly active enhancers were more prone to structural mutations (compared to point mutations) due to their open chromatin state that increases the possibility of genomic rearrangements. Juxtaposed with eQTLs and Hi-C approaches, such data can pinpoint genes with clinical implications (oncogenes, tumor-suppressor genes, tumor biomarkers) that are regulated by these enhancers [125]. For example, enhancers associated with expression of genes with clinical implications include enhancer 9 for PD-L1 which is a target for immunotherapy for melanoma and lung cancer [125,328]. Collectively, these studies spotlight the importance of regulatory element variants in cancer progression and propose combinational clinomic approaches that can lead to targets with clinical significance.

Current steps towards personalized treatment include patient categorization based on cancer-driver mutations in protein coding genes, with novel therapy trials like NCI-MATCH and SHIVA highlighting the importance for precision oncology [329,330,331]. Although these kinds of approaches seem to have encouraging preliminary results, there are limitations that mainly underlie the specificity of drug combinations [332]. This specificity depends on classification of patients based on the genomic profile, which demands cancer- and tissue-specific polymorphisms that confront treatment side effects or medicine inefficiency [333]. The regulatory role of non-coding variants comes to fill the gap of these restrictions as they can specify the patient drug-response [334]. For example, in breast cancer, patients carrying LINK-A rs12095274 A allele are prone to develop resistance to AKT inhibitors when compared to patients with C allele [335]. Focusing on regulatory non-coding variants that affect TF binding sites such as NFKB and STAT3 can also provide novel therapeutic approaches [336]. It is imperative to continue unveiling the function of non-coding variants independently or alongside with their associated ncRNAs not only to ensure risk assignment for genetic predisposition to the disease but also to tailor existing and future therapies to individual cancer genomes.

## 7. Conclusions and Future Perspectives

Genome instability and mutation is an essential feature of carcinogenesis that was first added in the cancer hallmark framework in 2011 [337]. It is undoubtedly a main characteristic that underlies the rapid evolution of cancer genomes during progression of the disease. Characterization of genetic variants with cancer development association can provide information on population risk stratification and prioritize the subgroups within a population for monitoring and primary prevention [338]. Combined with additional molecular biomarkers, determination of variant’s involvement in a cancer-related phenotype can be used as a predictor for the patient’s clinical outcome [339].

Uncovering the mutational landscape in cancer remains a huge challenge in the field of genomics. Tumor heterogeneity among patients with different genetic background in combination with the low conservation of non-coding sequences perplexes the discovery of their functional role during disease progression [67,340]. Although studies have shown a correlation of non-coding variants with predisposition and clinical outcome of the disease, the underlying mechanism of many variants is still unclear [341]. Moreover, discrimination between cancer-driver and passenger mutations adds another level of complexity in dissecting the mutational non-coding landscape [342]. Functional analysis of GWAS variants remains challenging [343], but novel techniques like the ones presented in this review can functionally dissect the role of non-coding regulatory variants involved in disease mechanisms. Future experimental efforts should also focus on dissection the role of non-coding variants in the biogenesis of miRNAs from lncRNA loci. By evaluating an individual’s risk of cancer development, their application for personalized prevention and monitoring programs will be enabled [344]. Should a person succumb to the disease, clarification of their genetic profile will enable risk assessment as a basis for personalized treatment [345]. In the era of precision medicine, where mutation and variation information are a top priority, investigating the role of non-coding genetic variants in regulating cancer cell function is of outmost importance [346]. This can lead to the detection of suitable and non-invasive molecular biomarkers for disease predisposition [347].

When it comes to treatment approaches in cancer, existing studies that focus on protein-coding genes, provided promising results. Parallel to their coding counterparts, ncRNAs are rising as more accessible candidates for pharmaceutical intervention [348]. In comparison to pharmaceutical molecules and antibodies used in the clinical act, there are no reports stating that the cancer cells show resistance to non-coding RNA therapy, while chemical modification of the ncRNAs further improve the half-time of ncRNA drugs compared to the other approaches [349,350]. For example, administration of miRNA molecules (common targets of TncV) has already given hopeful results in preclinical trials, by utilization for pathological left ventricular hypertrophy in mice [351].

The field of RNA-based therapeutics has yet to overcome multiple obstacles, such as the delivery system, target specificity and immunogenicity [352]. One of the biggest concerns is the identification of the best (and most functional) target, as most ncRNAs are not extensively characterized. Utilization of a miRNA-based cancer treatment is not yet applied because a miRNA might have different targets in various cell types which are not characterized, thus leading to off-target effects. Identification of a ncRNA’s mechanism in cancer alongside with its associated genetic variation remains a priority, which can be tackled by utilizing novel, RNA-centric methods [352,353,354]. Therefore, focusing our efforts on understanding and uncovering the non-coding portion of cancer variability not only can accommodate early diagnosis of malignancies but can also lead to the development of personalized therapeutic strategies.

## Figures and Tables

**Table 1:** Structural and functional genetic diversity in cancer.

Non-Coding Type	Variant ID	Target Locus	Mechanism	Cancer Type	Citation
Non-transcribed regulatory variants					
Promoters	rs11672691	PCAT19 promoter	NKX3.1, YY1 binding	Prostate	[68]
	rs887391	PCAT19 promoter	NKX3.1, YY1 binding	Prostate	
	rs17079281	DCBLD1 promoter	YY1 binding	Lung	[69]
	rs2267531	Glypican-3 promoter	-	HCC	[70]
	rs2280059	HSPH1 promoter	HSPH1 increased expression	NSCLC	[71]
Enhancer	rs11672691	PCAT19 Enhancer	NKX3.1, YY1, HOXA2 interaction with PCAT19	Prostate	[68]
	rs7463708	PCAT1 Enhancer	ONECUT, AR interaction with PCAT1	Prostate	[72]
	rs35252396	Enhancer between MYC and PVT1 genes	Binding of HIFs	RCC	[73]
	rs6983267	Enhancer between MYC and PVT1	Binding of HIFs	Prostate, Colorectal	[74]
	EGLN2 CNV	Enhancer	Genomic deletion	Ovaries	[75]
	rs67311347	Enhancer of ENTPD3-AS1	Binding of ZNF8	RCC	[76]
	rs4693608	Enhancer of HPSE	Regulation of HPSE	ALL	[77]
Silencer	rs249473	Silencer in AKT locus	Binding of AKT	Endometrial	[78]
Insulator	rs3850997	Insulator at GCLET intron	CTCF binding	Gastric	[79]
	MYCN CNV	Insulator of MYCN	Deletion, Loss of CTCF binding	Neuroblastoma	[80]
Transcribed regulatory variants					
miRNA	rs683/rs910 SNPs	3′UTR region of TYRP1	miRNA targeting	Μelanoma	[81]
	rs713065	miR-204	miRNA targeting of FZD4	NSCLC	[82]
	rs1071738	3′UTR of Palladin	miR-96/miR-182 targeting of Palladin	Breast	[83]
	rs1048638	3′UTR of CA9	miR-34a targeting of CA9	HCC	[84]
	rs928508	miR-30c	pri-mir-30c-1 biogenesis miR-30c interaction with SRSF3	Breast, Gastric	[85,86]
	rs6983267	Pre-miR-1307	pre-miR-1307 maturation	Colorectal	[87]
	rs11671784	Maturation process of miR-27a	miR-27a HOXA	Gastric	[88]
lncRNA	rs6983267	CCAT2	lncRNA interaction with CFIms25	Colorectal	[89]
	rs114020893	lncRNA NEXN-AS1	LncRNA secondary structure	Lung	[90]
	rs664589	miR-194-5p	miR-194-5p interaction with MALAT1	Colorectal	[91]
	rs1317082	CCSlnc362	miR-4658 interaction with CCSlnc362	Colorectal	[92]
	rs11752942	LINC00951	miRNA-149 interaction with LINC00951	ESCC	[93]
	rs11655237	LINC00673	miR-1231 interaction with LINC00673	PDCA	[94]
	rs10251977	EGFR-AS1	Isoform selection via miR-891b and EGFR-AS interaction	Oral	[95]

**Table 2:** State-of-the art methodologies for functional genomic variant identification. Access: 15 July 2021.

Experimental Approach	Advantages	Disadvantages	Publicly Available Databases/Software
Methodologies to study genomic areas in open-chromatin state			
DNase-seq	• Enrich in cis-acting Res • No need for specific TF targeting • scDNase-seq improves sensitivity	• Biased in favor of promoters	• HOMER (Hypergeometric Optimization of Motif EnRichment) http://homer.ucsd.edu/homer/download.html
FAIRE-seq	• Simple application • Low bias • Sensitivity for intronic	• Low signal-to-noise ratio. • Requires high fixation efficiency.	• ENCODE: Wiggler https://sites.google.com/site/anshulkundaje/projects/wiggler
MNase-seq	• Less noise from mtDNA	• Laborious protocol • Digestion-based	• http://compbio-zhanglab.org/NUCOME/
ATAC-seq	• Efficiency • Simple, cost-efficient application • Nucleosome and TF occupancy	• Demands coupling with other techniques	• ENCODE-DCC version 10 https://github.com/ENCODE-DCC/encoded/releases/tag/v101.0
Methodologies for non-transcribed functional variant identification			
MPRAs/CRE-seq	• High-throughput examination of enhancer activity • Allows multiple independent examinations	• Episomal assay • Cell-type specific enhancer activation profile • False-positive ratio	• Shendurelab/MPRAflow https://github.com/shendurelab/MPRAflow
STARR-seq	• High-throughput examination of enhancer activity • Reduced false-positive ratio • No barcoding	• Episomal assay • Cell-type specific enhancer activation profile • Reporter transcript stability	• Gersteinlab/starrpeaker https://github.com/gersteinlab/starrpeaker • hyulab/eSTARR https://github.com/hyulab/eSTARR
ChIA-PET	• Precise global interaction map • Long-read ChIA-PETS has improved mapping efficiency	• Complex data analysis • Inefficient • Demands coupling with RNA-targeted methodology	• ChIA-PET Utilities-CPU https://github.com/cheehongsg/CPU • Mango https://github.com/dphansti/mango • TheJacksonLaboratory/ChIA-PIPE https://github.com/TheJacksonLaboratory/ChIA-PIPE
HiChIP	• Efficiency • Low false-positive ratio • Simple workflow	• Not available	• FitHiChIP https://github.com/ay-lab/FitHiChIP
PLAC-seq	• Efficiency • Specificity • Simple workflow	• Not available	• HPRep https://github.com/yunliUNC/HPRep • MAPS https://github.com/HuMingLab/MAPS
ChIRP-seq	• Commonly used	• Increased false-positive ratio • Targets known RNA	• Not available

**Table 3:** Methodologies used to study the effect of variants in transcript-based regulatory networks. Access: 15 July 2021.

Experimental Approach	Advantages	Disadvantages	Publicly Available Databases/Software
Methodologies for transcribed functional variant identification			
RAP-seq	• Genome-wide RNA:DNA interaction maps • Low background noise	• Known RNA sequence	• SPRITE https://github.com/GuttmanLab/sprite-pipeline
RNP-MaP	• Efficient • Resolution • Unbiased • Study protein networks	• Coupling with protein-targeted methodology	• Not available
CARPID	• Specificity • Low background noise • Determine allelic expression	• Coupling with protein-targeted methodology	• Not available
PTRE-seq	• High-throughput examination of 3′UTR regulatory activity	• Not available	• Not available
PARS-seq	• RNA structural information • Distinguish paired/unpaired bases • Alternative to MS, NMR, crystallography	• Non-specific enzyme digestion • RNA over-digestion • Need for optimization • Only in vitro applications	• RNAFramework https://github.com/dincarnato/RNAFramework

**Table 4:** CRISPR-Cas systems utilized in functional non-coding variant validation. Access: 15 July 2021.

Experimental Approach	Advantages	Disadvantages	Publicly Available Databases/Software
CRISPR-Cas Systems	• Allows variant correction/creation • Low off-target effects (especially when using nickase Cas9) • Activation and inhibition of regulatory element function • Alteration in methylation status • RNA targeting	• Needs fine-tuning to avoid off-target effects • Efficiency differs between systems	• Design http://www.rgenome.net/be-designer/ http://zifit.partners.org/ZiFiT/ http://www.e-crisp.org/E-CRISP/ https://chopchop.cbu.uib.no/ http://crispr-era.stanford.edu/ https://portals.broadinstitute.org/gpp/public/analysis-tools/sgrna-design • Analysis http://www.rgenome.net/be-analyzer/#

**Table 5:** Summary of non-coding variants associated with cancer clinomics.

SNP ID	Target Locus	Clinical Trait	Cancer Type	Citation
rs291593	DPYD 3′UTR	Drug toxicity	Breast cancer	[316]
rs3209896, rs1824125	AKR1C3 3′UTR, PGR 3′UTR	Progression-free Survival		
rs1054899	ALDH5A1 3′UTR	Chemotherapeutic response to FAC		
rs7756222, rs9487402	SLC22A16 3′UTR	Overall survival		
rs13230517	RP11-3N2.1 promoter	Cancer Risk	Colorectal cancer	[317]
rs531564	pri-miR-124	Lymph node metastasis		
rs3741219, rs2910164, rs4938723	H19 lncRNA, hsa-mir-146a, hsa-mir-34b/c	Cancer risk	Hepatocellular carcinoma	[318]
rs11614913, rs2292832	hsa-mir-196a-2, hsa-mir-149	Cancer risk	HBV-related HCC	[319]

## Data Availability

Not applicable.
